# Alignment strategy for different types of varus knee with generic instruments: Mechanical alignment or kinematic alignment?

**DOI:** 10.1186/s13018-023-04257-8

**Published:** 2023-10-28

**Authors:** Haoran Lin, Qi Cheng, Guangjian Li, Jie Zhao, Qiang Wang

**Affiliations:** 1 Department of Orthopaedic Surgery, The First Affiliated Hospital of Wannan Medical College, No. 2 Zheshan West Road, Wuhu, 241001 Anhui Province People’s Republic of China; 2 Psychiatry and Psychology Department, Changzhou Dean Hospital, Changzhou, 213003 Jiangsu Province People’s Republic of China

**Keywords:** Total knee arthroplasty, Mechanical alignment, Kinematic alignment, Varus knee, Generic instruments

## Abstract

**Objective:**

A thorough examination of the available approaches is crucial to comprehensively understand the variance among the alignment strategies employed in total knee arthroplasty (TKA). In this study, we assessed the functional outcomes during the perioperative and postoperative periods of TKA in patients using generic instruments with varus knee to compare the mechanical alignment (MA) and kinematic alignment (KA) procedures.

**Methods:**

A total of 127 patients from the First Affiliated Hospital of Wannan Medical College who had undergone unilateral TKA between November 2019 and April 2021 were included. The patients with varus knee deformity were categorized into two groups [type I (*n* = 64) and type IV (*n* = 63)] based on the modified coronal plane alignment of the knee (mCPAK) classification. The type I and IV groups were further subdivided into MA (*n* = 30 and *n* = 32) and KA subgroups (*n* = 34 and *n* = 21), respectively. The clinical information collected included sex, surgical side, age, body mass index, and perioperative data [including operation time, intraoperative blood loss, length of hospital stay, and the American Society of Anesthesiologists (ASA) classification]. All patients were monitored for 12 months post-surgery to evaluate the recovery of knee joint function. During this period, the Knee Disability and Osteoarthritis Outcome Score for Joint Replacement (KOOS JR) and the active range of motion (AROM) and visual analog scale (VAS) pain scores were compared at different time points, i.e., before the operation and 6 weeks, 6 months, and 12 months post-operation. Additionally, the patients’ subjective experiences were assessed at 6 and 12 months post-surgery using Forgotten Joint Score Knee (FJS-12 Knee), while complications were recorded throughout the monitoring period.

**Results:**

No significant variances were observed in ASA classification, operation duration, blood loss volume during surgery, and hospital stay length between the patients who underwent KA TKA and those who received MA TKA (*P* > 0.05). During the initial 6 weeks post-operation, the KA group exhibited a significantly reduced average VAS pain score (*P* < 0.05), with no such differences at 6 months and 1 year after the surgery (*P* > 0.05). Furthermore, the KA group had significantly higher scores on the KOOS JR at 6 weeks, 6 months, and 1 year following the surgery (*P* < 0.05). Moreover, the AROM score of the KA group significantly improved only at 6 weeks after the surgery (*P* < 0.05); however, no prominent differences were found at 6 months and 1 year after the operation (*P* > 0.05). The KA cohort also exhibited a significant increase in FJS-12 Knee at 1 year following the operation (*P* < 0.05), whereas no such difference was detected at 6 months following the surgery (*P* > 0.05). Thus, compared to the MA method, the KA procedure provided pain relief and improved active motion range within 6 weeks after the surgery in patients undergoing TKA. Further, the KOOS JR exhibited significant increases at 6 weeks, 6 months, and 1 year while the FJS-12 Knee demonstrated a significant increase at 1 year after the KA TKA procedure.

**Conclusion:**

Therefore, our study results suggest that the KA approach can be considered in patients using generic instruments with varus alignment of the knee, particularly those with mCPAK type I and IV varus knees, to help improve patient satisfaction.

## Introduction

From its inception, the mechanical alignment (MA) method has been consistently considered the benchmark for total knee arthroplasty (TKA), achieving an excellent success rate over the years [1]. The establishment of a neutral mechanical axis and a horizontal joint line via MA is a well-known optimal mechanical setting for ensuring implant longevity. However, MA does not address the natural differences in coronal alignment among individuals [2–4], and this “one-size-fits-all” method can result in considerable biomechanical consequences and a high rate of patient dissatisfaction [5]. Approximately 20% of patients without any other issues have been reported to express dissatisfaction [6–8]. Moreover, over 50% of patients experience residual symptoms [9], while up to 25% show hesitancy to receive additional surgery [10]. Compared to individuals with MA TKA treatment, those who undergo kinematic alignment (KA) TKA demonstrate notable improvements in pain relief, functionality, flexion, and a heightened feeling of a natural knee [11–15]. However, KA TKA is accompanied by increased costs related to navigation, 3D printing, and robotic systems [16–23]. Therefore, this study utilized Howell’s calipered technique, i.e., a technique in which each step is controlled by caliper measurement of the resected bone and chondral structures [24, 25]. This intervention is a simple, economical, fast, and a highly reproducible surgical approach that does not require expensive equipment used in KA TKA [26–29]. Although many studies have compared MA with KA [11–13, 30, 31], there are still not many researches using traditional tools [26, 28], especially no research has assessed the different alignment methods used to treat the varying types of varus knees. In 2021, MacDessi et al. proposed the coronal plane alignment of the knee (CPAK), which simplified the classification to nine knee phenotypes by including only two key variables: arithmetic hip–knee–ankle (aHKA) and joint line obliquity (JLO) [32]. In the following year, Hsu et al. proposed the modified coronal plane alignment of the knee (mCPAK) classification, in which a new variable called “actual JLO” (aJLO) was introduced, which reflects the degree to which the JLO is parallel to the ground. aJLO is described by the equation aJLO = 90° − (LDFA + MPTA)/2. aHKA was determined by MPTA-LDFA to predict structural alignment of the lower extremity prior to arthritis. Modifying the CPAK classification by changing the neutral boundary of aHKA to 0° ± 3° and using aJLO as a new variable corrected the uneven distribution when applying the CPAK classification in Asian populations [33]. In this study, we evaluated the initial effectiveness of MA and KA TKAs in patients with type I and IV varus knees according to the modified coronal plane alignment of the knee (mCPAK) classification developed by Hsu et al. as a guiding framework [33].

## Materials and methods

### Patient selection

A total of 127 patients who underwent primary TKA at the First Affiliated Hospital of Wannan Medical College between November 2019 and April 2021 were selected.

### Inclusion criteria

The inclusion criteria were as follows: patients who had undergone unilateral TKA, classification of the varus knee into type I or IV based on the mCPAK classification, clear indications for surgery without any apparent surgical contraindications, and surgery by skilled doctors.

### Exclusion criteria

Patients were excluded if they met any of the following conditions: post-traumatic or septic arthritis of the knee, previous knee surgery, a body mass index (BMI) of > 40 kg/m^2^, valgus knee, contralateral TKA or ipsilateral total hip arthroplasty, or < 1 year of follow-up.

### General information

From November 2019 and April 2021, 127 patients underwent unilateral TKA at our hospital. We collected the data of gender, age, and BMI as well as anteroposterior and lateral knee X-rays and full-length anteroposterior X-rays of the lower limbs acquired pre- and post-surgery. Among the total patients, 47 were men, and 80 were women. Additionally, 56 underwent surgery on their left knee, whereas 71 had surgery on their right knee. The patients’ ages ranged from 49 to 83 years, with an average of 66.54 years. Under the mCPAK categorization criteria for varus knee, 64 patients were classified into type I, and 63 were categorized as type IV. Among those with type I varus knee, the MA and KA techniques were used in 30 and 34 patients, respectively. In the case of the patients with type IV varus knee, 32 underwent the MA procedure, while 32 were aligned using the KA method. All surgeries were performed by experienced orthopedic surgeons. Based on the above differentiation, the study patients were categorized into the following four groups: type I-MA, type I-KA, type IV-MA, and type IV-KA. No significant group differences were found in age, gender, surgical side, American Society of Anesthesiologists (ASA) classification (we have consulted the website https://www.asahq.org/standards-and-practice-parameters/statement-on-asa-physical-status-classification-system on May 3, 2021), or BMI (*P* > 0.05). Similarly, no significant differences were detected in surgical duration, volume of blood lost during surgery, and length of hospital stay among the four groups (*P* > 0.05) (Table 1).

**Table 1:** Comparison of general and perioperative patient data

Category	I-MA	I-KA	IV-MA	IV-KA	*χ*^2^/*F* value	*P*-value
*n*	30	34	32	31		–
Sex Male/female	10/20	12/22	13/19	12/19	0.397	0.941
Side, left/right	13/17	15/19	14/18	14/17	0.023	0.999
Age (year)	66.20 ± 7.41	66.88 ± 7.42	65.94 ± 7.18	67.10 ± 7.24	0.179	0.911
ASA	1.27 ± 0.45	1.24 ± 0.43	1.19 ± 0.40	1.23 ± 0.43	0.183	0.908
BMI (kg/m^2^)	24.10 ± 2.56	23.27 ± 3.27	24.30 ± 2.36	23.79 ± 2.73	0.865	0.461
Operative time (min)	56.00 ± 4.01	57.29 ± 5.56	55.31 ± 3.64	55.61 ± 3.43	1.383	0.251
Intraoperative bleeding (ml)	114.97 ± 9.33	116.68 ± 11.49	113.25 ± 7.37	116.97 ± 8.87	1.067	0.366
Hospital stay (day)	7.83 ± 1.23	7.88 ± 1.33	8.13 ± 1.16	7.90 ± 1.22	0.362	0.780

### Surgical technique

All TKA surgeries were conducted using a medial parapatellar approach with posterior-stabilized, bone-cemented, and fixed-bearing implants. The intervention was concealed from the patients and independent outcome assessors gathering the patient-reported outcome measures. Furthermore, every patient underwent an identical preoperative evaluation and pain management plan during the perioperative period to guarantee blinding. The MA group underwent a standard posterior cruciate ligament-substituting (PS) TKA via a measured resection technique using the Zimmer NexGen total knee implant, along with a tourniquet throughout the procedure. In this procedure, access to the medial patella was obtained through a midline incision in front of the knee. Initially, the tibia was incised, followed by the excision of the intercondylar eminence and anterior and posterior horns of the meniscus. Double hip hooks were employed to expose the medial tibial plateau, which was marked using an electrocautery device. Furthermore, a hook was inserted into the posterior tibial plateau and raised to expose the entire plateau. Next, an alignment guide was placed on the midpoint of the tibial intercondylar eminence, wherein the distal end extended to the second metatarsal, and the proximal aspect was two finger-breadths proximal to the distal aspect. Based on the extramedullary alignment, the mechanical axis was referenced for tibial cutting, ensuring precise anterior–posterior angulation and a posterior slope of 7°. The femur was then cut with a 6° valgus and 3° external rotation according to the femoral implant size or referencing the epicondylar axis. After the flexion–extension gap and medial–lateral balance were evaluated, gap balance was achieved through adequate soft tissue release and bone removal. Next, the trial implant was installed, and the flexion–extension movement, stability, and patellar tracking were examined. The implant was finally fixed with bone cement after pulse lavage. Patellar reshaping was performed in all patients, whereas patellar replacement was not required in all of them. A pain-relieving fluid mixture (200 mg of ropivacaine, 5 mg of morphine, and 1 ml of compound betamethasone diluted to 100 ml using saline) was injected into the surrounding joint capsule via a multipoint injection technique. After the closure of the deep fascia, a tranexamic acid solution (1.5 g of tranexamic acid in 50 ml of saline) was administered into the joint capsule, followed by drain insertion in all patients.

In the case of the KA group, also using the Zimmer NexGen PS total knee implant, adherence to the principle that the kinematic alignment of the femoral and tibial components was consistent with the native joint line [25].

First, consider the femoral component. The femoral implant needs to be aligned with the three kinematic axes that guide knee kinematics in order to achieve a good functional outcome, which is the cornerstone of the KA technique. The reliability of the femoral implant positioning comes from two main aspects: First, the bicondylar cartilage thickness is fairly standard and similar, averaging about 2 mm [34]. By using a spacer of 2-mm thickness on the wear side [24], it was placed medially as our inclusion criterion was varus knee. Secondly, in the KA technique, the osteotomy thickness can be easily predicted without any complex preoperative planning because the sum of “osteotomy thickness plus cartilage wear (2 mm) plus kerf thickness (approximately 1 mm)” should equal the thickness of the implant. Therefore, measurement of osteotomy thickness is crucial in the KA technique for detection and do corrections if necessary [24]. It is worth special consideration that the KA technique selects neutral posterior referencing guide, noting the difference from the 3° external rotation of the MA technique, due to the fact that typical posterior femoral condylar cartilage wear in both varus and valgus osteoarthritic knees is less than 1 mm, which is clinically unimportant to correct. Calipers were used to measure the thickness of the osteotomies at different sites, including the distal medial and lateral femoral condyles and the medial and lateral posterior femoral condyles. After correcting for cartilage wear and kerf, the femoral implant conformed to the kinematic alignment when each osteotomy thickness was within ± 0.5 mm of the corresponding distal and posterior region of the condyle of femoral component [25].

Next, consider the tibial component. The tibial extramedullary guide was adjusted to anatomical varus, i.e., the cut plane of proximal tibial was adjusted to be parallel to the oblique plane of the tibial articulation that is naturally varus after the correction of wear. (Only patients with varus knees were included in this study) This was followed by the rotational alignment step of the tibial implant: The boundaries of oval-shaped lateral tibial condyle were outlined with a series of black dots (marked intraoperatively with an electrosurgical knife), a line was drawn on the A/P axis that bisects the oval shape, and two holes were drilled on the articular surface of the medial tibial condyle parallel to the A/P axis using a guide. On the resected surface of the tibial plateau, two A/P lines were drawn parallel to the two holes, which were drilled with pins for the clarification of their location. Make sure the A/P axis of the trial tibial component parallel to these two A/P lines [35]. Finally, the forward offset of the distal medial femoral condyle relative to the tibia was measured with calipers in the osteoarthritic knee and reconstructed knee with trial components, respectively, at 90° of knee flexion, where the amount of cartilage wear on the distal medial femoral condyle needed to be subtracted from the measurement to determine the normal offset. The posterior tibial slope was fine-tuned until the offset of the reconstructed knee with trial components matched that of the osteoarthritic knee [24]. The remaining surgical procedures were comparable to those performed in the MA group.

Postoperative management was standardized across the MA and KA groups, and the physical therapists were blinded to the intervention. The patient-reported outcomes were evaluated using the following measures: visual analog scale (VAS) score to measure pain levels at rest and during activity on a scale of 0–10 (0 = no pain and 10 = most severe pain), Knee Disability and Osteoarthritis Outcome Score for Joint Replacement (KOOS JR) [36] to assess knee joint damage and osteoarthritis (ranging from 0 to 100, with 0 = worst score and 100 = best score), Forgotten Joint Score Knee (FJS-12 Knee) [37] to determine patient satisfaction (ranging from 0 to 100, with 0 = worst score and 100 = best score), and active range of motion (AROM) score to estimate the knee joint function. Furthermore, surgery difficulty and patient recovery associated with the two alignment methods were compared by analyzing perioperative data, such as surgery duration, volume of blood loss during surgery, and length of hospital stay. The scores on the KOOS JR, VAS, and AROM were compared at different times, including before the surgery and at 6 weeks, 6 months, and 12 months post-surgery. The FJS-12 Knee was differentiated at 6 and 12 months following the surgery to evaluate postoperative subjective feeling in the patients of the two groups.

### Evaluation indicators

**Fig. 1:**
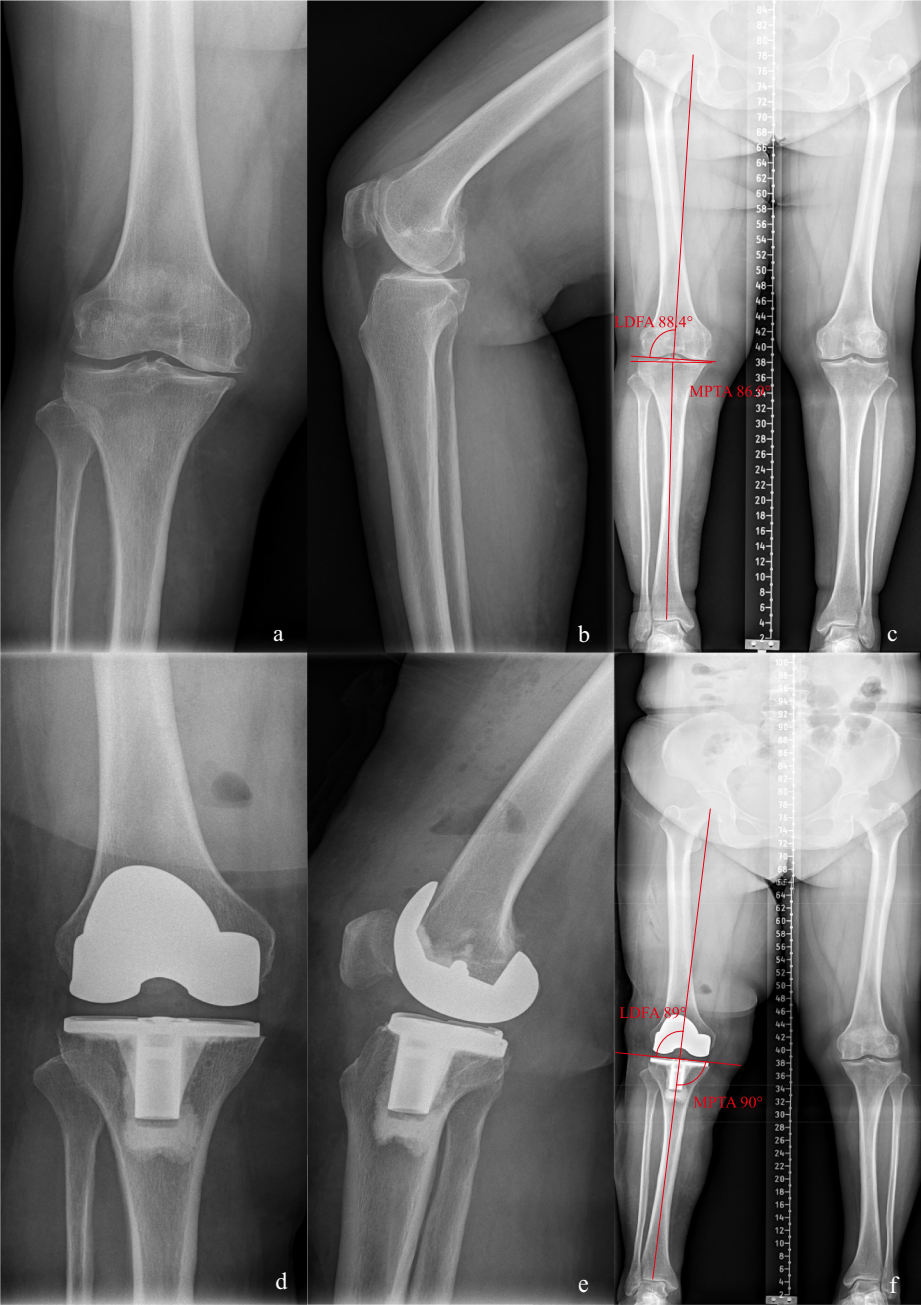
Case 1, preoperative and postoperative standard images of the right knee joint of a 63-year-old female patient with type I knee varus treated with MA-TKA are shown. Figures** a**,** b** and ** c** show preoperative standard images of the right knee joint from the front and side as well as full-length X-rays of the lower limbs, respectively; Figures** d**,** e** and **f** show postoperative standard images of the right knee joint from the front and side as well as full-length X-rays of the lower limbs, respectively. Figure** c** shows that the preoperative MTPA and LDFA angles were 86.9° and 88.4°, respectively, and Figure** f** shows that, following MA-TKA, the corrected MTPA and LDFA angles were 90° and 89°, respectively

**Fig. 2:**
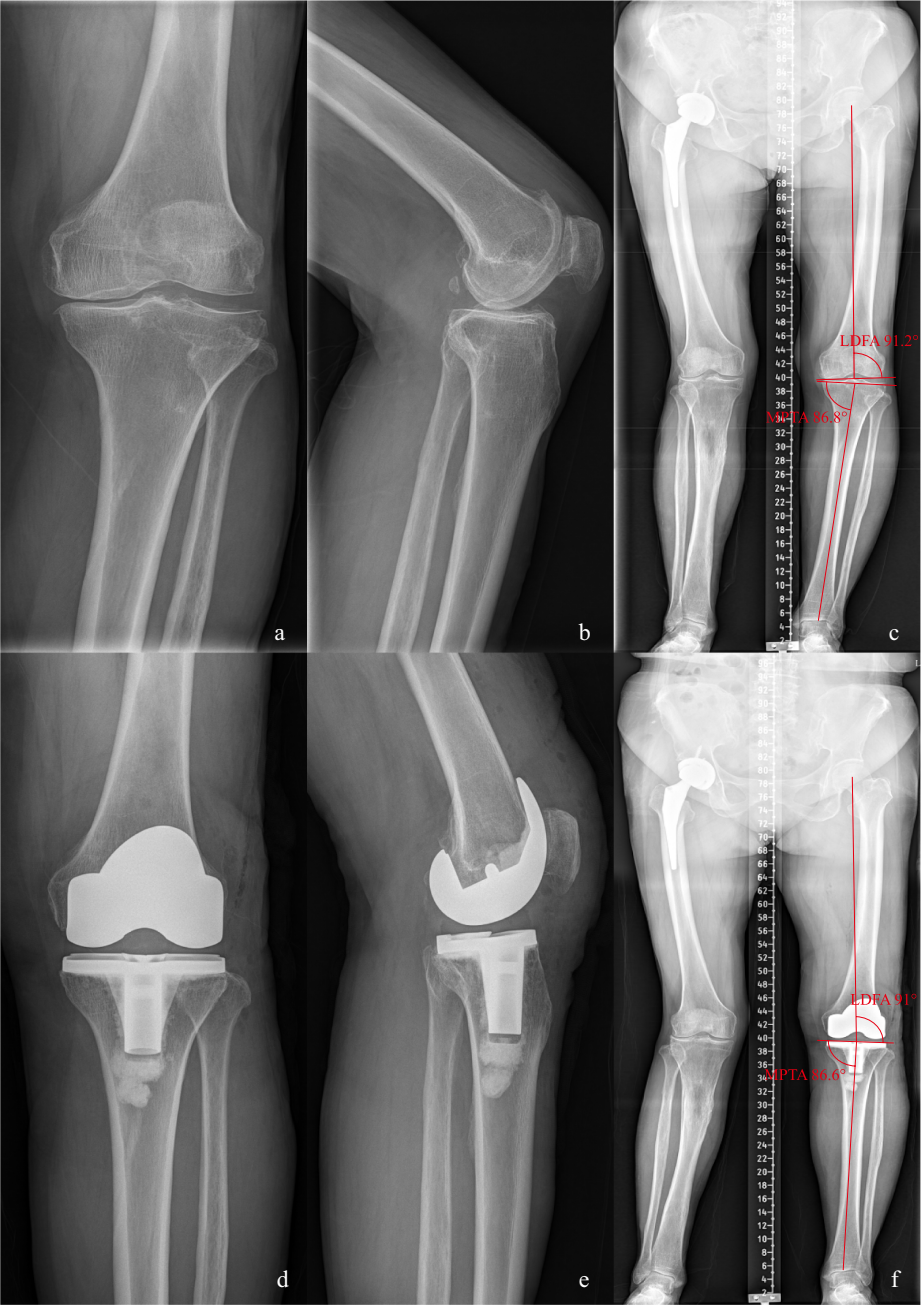
Case 2, preoperative and postoperative standard images of the left knee joint of a 68-year-old female patient with type I knee varus treated with KA-TKA are shown. Figures** a**,** b**, and** c** show preoperative standard images of the left knee joint from the front and side as well as full-length X-rays of the lower limbs, respectively; Figures** d**,** e** and** f** show postoperative standard images of the left knee joint from the front and side as well as full-length X-rays of the lower limbs, respectively. Figure** c** shows that the preoperative MTPA and LDFA angles were 86.8° and 91.2°, respectively, and Figure** f** shows that, following KA-TKA, the corrected MTPA and LDFA angles were 86.6° and 91°, respectively

**Fig. 3:**
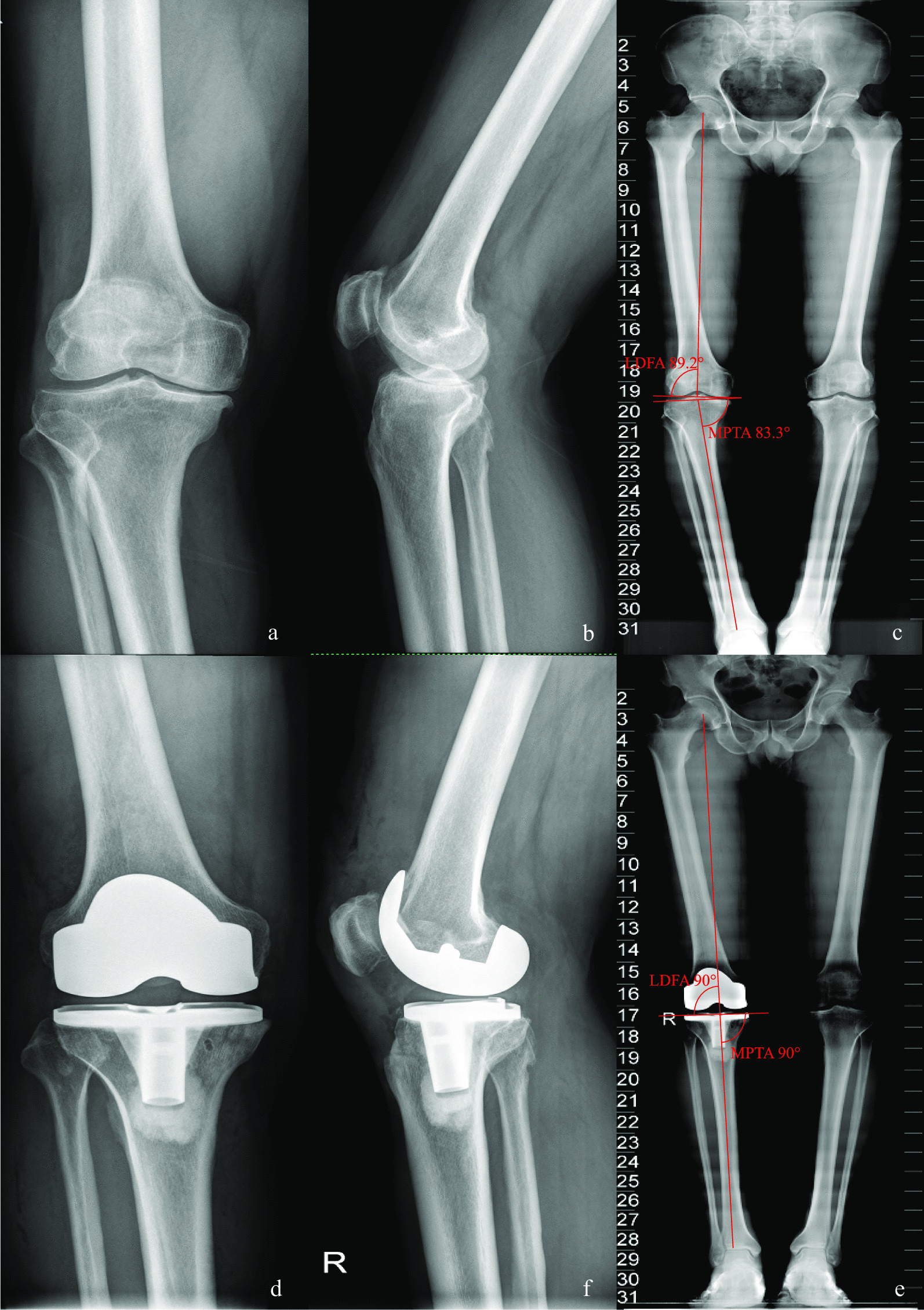
Case 3, preoperative and postoperative standard images of the right knee joint of a 61-year-old male patient with type IV knee varus treated with MA-TKA are shown. Figures** a**,** b**, and** c** show preoperative standard images of the right knee joint from the front and side as well as full-length X-rays of the lower limbs, respectively; Figures** d**,** e** and** f** show postoperative standard images of the right knee joint from the front and side as well as full-length X-rays of the lower limbs, respectively. Figure** c** shows that the preoperative MTPA and LDFA angles were 83.3° and 89.2°, respectively, and Figure** f** shows that, following MA-TKA, the corrected MTPA and LDFA angles were 90° and 90°, respectively

**Fig. 4:**
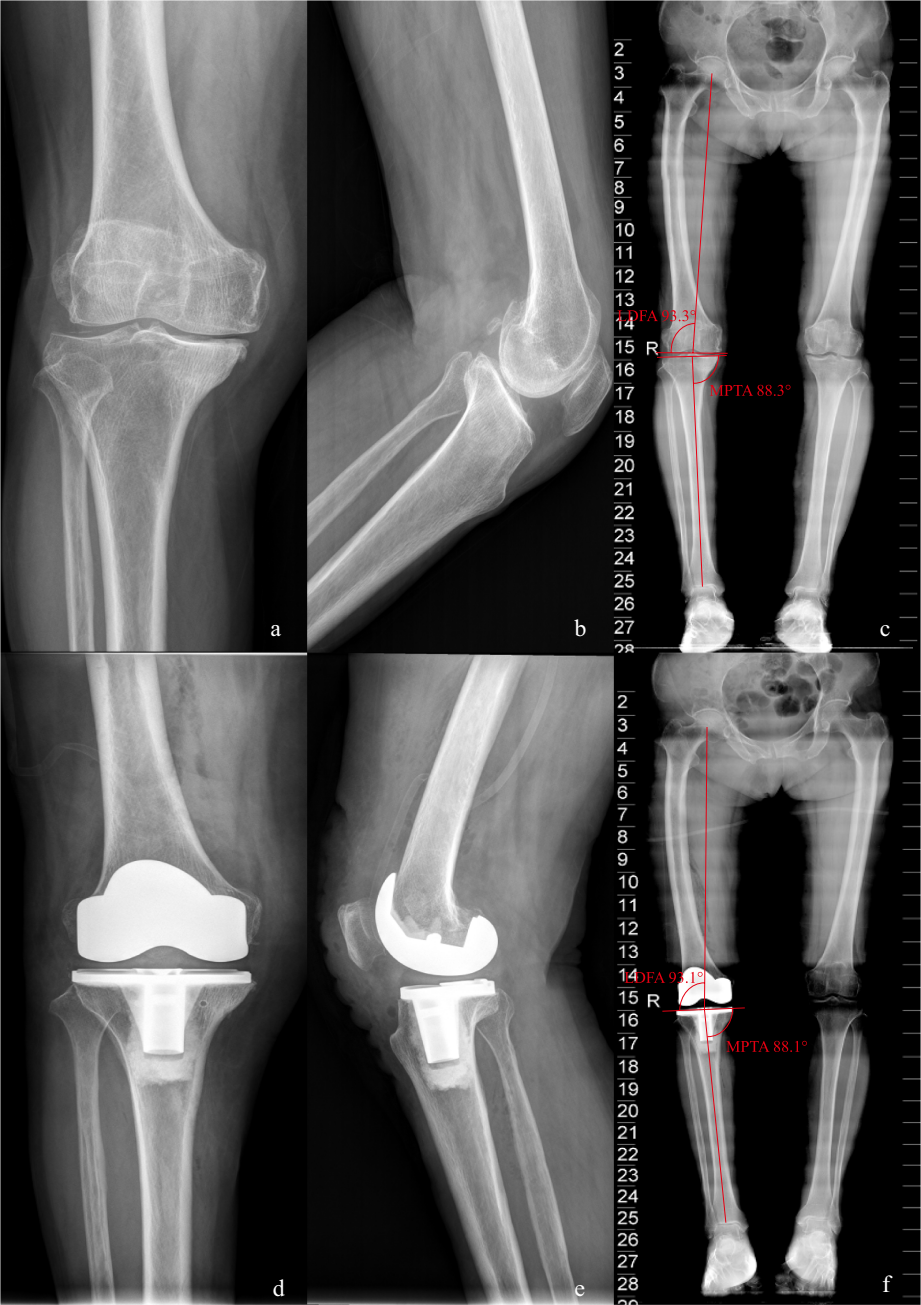
Case 4, preoperative and postoperative standard images of the right knee joint of a 78-year-old female patient with type IV knee varus treated with KA-TKA are shown. Figures **a**,** b**, and** c** show preoperative standard images of the right knee joint from the front and side as well as full-length X-rays of the lower limbs, respectively; Figures **d**,** e** and** f** show postoperative standard images of the right knee joint from the front and side as well as full-length X-rays of the lower limbs, respectively. Figure** c** shows that the preoperative MTPA and LDFA angles were 88.3° and 93.3°, respectively, and Figure** f** shows that, following KA-TKA, the corrected MTPA and LDFA angles were 88.1° and 93.1°, respectively

The evaluation was conducted based on the standard images of the knee joint from the front and side as well as full-length X-rays of the lower limbs using a large flat-panel multifunctional digital CT (i.e., computed tomography) system (Toshiba Aquilion TSX-101A). The built-in measurement method of the hospital’s picture archiving and communication system was utilized to evaluate the images (Figs. 1, 2, 3, and 4).

### Statistical analysis

All statistical analyses were conducted using IBM SPSS 25.0 software (USA). Continuous variables were reported as mean ± standard deviation, and the between-group comparisons for categorical variables (e.g., gender and affected side) were evaluated using the Chi-squared test. Additionally, one-way analysis of variance (ANOVA) was used to compare the continuous variables (e.g., age, BMI, surgery duration, volume of intraoperative blood loss, and hospital stay length) among groups. The PROMs were assessed using the scores obtained on the VAS, KOOS JR, AROM, and FJS-12 Knee. Two-way repeated measures ANOVA was used to estimate the changes in these measures from the preoperative period to 1-year follow-up. Statistical significance was set at a *P*-value of < 0.05.

## Results

**Fig. 5:**
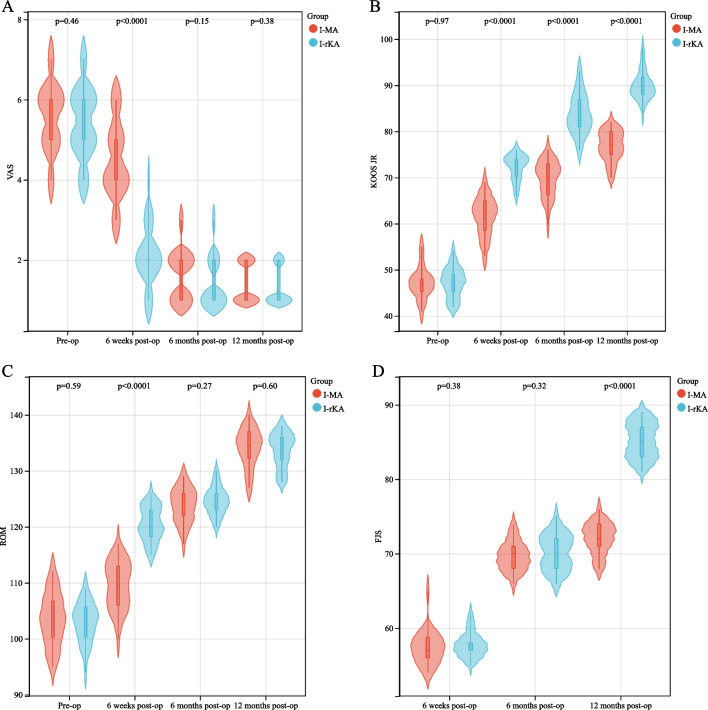
Comparisons of scores of patients with type I knee varus treated with MA-TKA and KA-TKA are shown. Figure** a**,** b**, and** c** show the comparisons of VAS, KOOS JR, and AROM scores of patients with type I knee varus treated with MA-TKA and KA-TKA at different time points, i.e., before the operation and 6 weeks, 6 months, and 12 months post-operation, respectively. Figures** d** shows the comparison of FJS-12 Knee of patients with type I knee varus treated with MA-TKA and KA-TKA at different time points, i.e., 6 weeks, 6 months, and 12 months post-operation. Abbreviation: VAS, visual analog scale; KOOS JR, Osteoarthritis Outcome Score for Joint Replacement; AROM, active range of motion; FJS-12 Knee, Forgotten Joint Score Knee

**Fig. 6:**
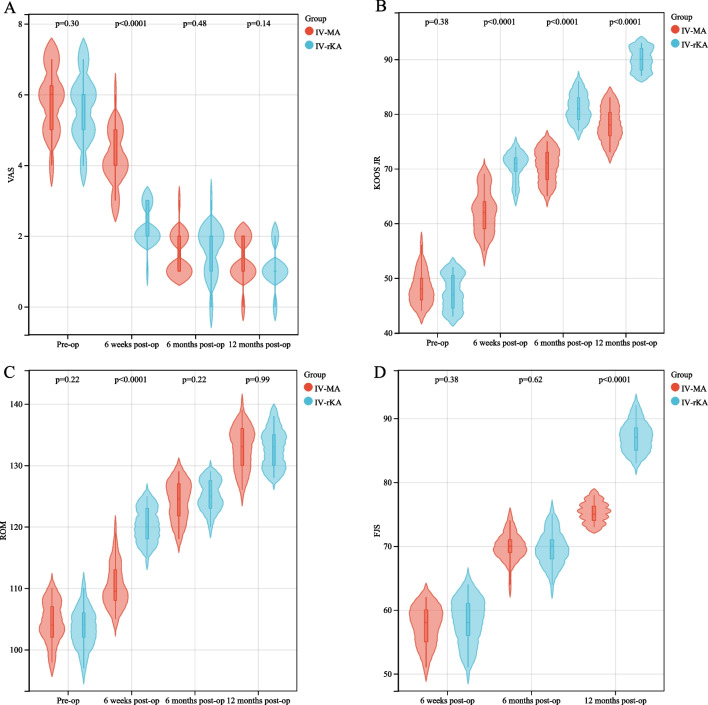
Comparisons of scores of patients with type IV knee varus treated with MA-TKA and KA-TKA are shown. Figures** a**,** b**, and** c** show the comparisons of VAS, KOOS JR, and AROM scores of patients with type IV knee varus treated with MA-TKA and KA-TKA at different time points, i.e., before the operation and 6 weeks, 6 months, and 12 months post-operation, respectively. Figure ** d** shows the comparison of FJS-12 Knee of patients with type IV knee varus treated with MA-TKA and KA-TKA at different time points, i.e., 6 weeks, 6 months, and 12 months post-operation. Abbreviation: VAS, visual analog scale; KOOS JR, Osteoarthritis Outcome Score for Joint Replacement; AROM, active range of motion; FJS-12 Knee, Forgotten Joint Score Knee

The varus knee deformities were compared within the type I-MA and -KA groups as well as within the type IV-MA and -KA groups. At 6 weeks after the operation, the KA group reported a lower VAS pain score than the MA group (*P* < 0.05). However, no significant variance was observed in the VAS pain ratings between the two cohorts at the 6-month and 1-year follow-up periods (*P* > 0.05). At 6 weeks, 6 months, and 1 year after the operation, the KA group demonstrated a significantly higher KOOS JR, indicating better function and pain levels than the MA group (*P* < 0.05). At 6 weeks post-surgery, the KA group exhibited a significantly greater AROM score than the MA group (*P* < 0.05). Nevertheless, no such differences were found in the AROM scores between the two groups during the 6-month follow-up (*P* > 0.05). At the 1-year follow-up, the KA group exhibited a significantly higher FJS-12 Knee than the MA group (*P* < 0.05). However, the FJS-12 Knee between the two groups did not show any significant variation during the 6-month postoperative follow-up (*P* > 0.05) (Tables 2 and 3, Figs. 5 and 6).

**Table 2:** Two-factor repeated measures ANOVA for the postoperative scores of patients diagnosed with type I varus knee

Group	Time	VAS	KOOS	AROM	FJS-12 Knee
I-MA (*n* = 30)	T1: preoperative	5.56 ± 0.81	47.20 ± 3.39	103.53 ± 4.25	
	T2: 6 weeks postoperative	4.46 ± 0.93	61.60 ± 4.14	109.53 ± 5.65	57.33 ± 2.19
	T3: 6 months postoperative	1.63 ± 0.61	69.80 ± 3.84	123.87 ± 3.05	69.67 ± 1.94
	T4: 1 year postoperative	1.40 ± 0.49	76.83 ± 3.23	134.13 ± 3.42	72.07 ± 2.08
I-KA (*n* = 34)	T1: preoperative	5.41 ± 0.85	47.23 ± 3.21	102.97 ± 3.50	
	T2: 6 weeks postoperative	2.08 ± 0.71	72.26 ± 2.71	120.50 ± 3.04	57.76 ± 1.63
	T3: 6 months postoperative	1.41 ± 0.60	77.25 ± 8.14	124.65 ± 2.42	70.21 ± 2.38
	T4: 1 year postoperative	1.29 ± 0.46	89.94 ± 2.99	133.71 ± 3.06	85.21 ± 2.35
Overall comparison	HF correction factor	0.883	0.979	0.970	1.000
	*F*, *P*-value between groups	51.370, *P* < 0.001	531.66, *P* < 0.001	33.541, *P* < 0.001	277.54, *P* < 0.001
	*F*, *P*-value within group	525.464, *P* < 0.001	1227.92, *P* < 0.001	956.597, *P* < 0.001	1494.094, *P* < 0.001
	Interaction *F*, *P*-value	43.516, *P* < 0.001	51.189, *P* < 0.001	43.010, *P* < 0.001	177.536, *P* < 0.001
Refined comparison between groups (*LSD-t* test, *P*-value)	T1	*P* = 0.464	*P* = 0.966	*P* = 0.585	
	T2	*P* < 0.001	*P* < 0.001	*P* < 0.001	*P* = 0.371
	T3	*P* = 0.153	*P* < 0.001	*P* = 0.259	*P* = 0.329
	T4	*P* = 0.381	*P* < 0.001	*P* = 0.600	*P* < 0.001
Refined comparison within group (*LSD-t* test, *P*-value)	I-MA group: T2 versus T1	*P* < 0.001	*P* < 0.001	*P* < 0.001	*P* < 0.001
	I-MA group: T3 versus T1	*P* < 0.001	*P* < 0.001	*P* < 0.001	*P* < 0.001
	I-MA group: T4 versus T1	*P* < 0.001	*P* < 0.001	*P* < 0.001	*P* < 0.001
	I-MA group: T3 versus T2	*P* < 0.001	*P* < 0.001	*P* < 0.001	*P* < 0.001
	I-MA group: T4 versus T2	*P* < 0.001	*P* < 0.001	*P* < 0.001	*P* < 0.001
	I-MA group: T4 versus T3	*P* = 0.031	*P* < 0.001	*P* < 0.001	*P* < 0.001
	I-KA group: T2 versus T1	*P* < 0.001	*P* < 0.001	*P* < 0.001	*P* < 0.001
	I-KA group: T3 versus T1	*P* < 0.001	*P* < 0.001	*P* < 0.001	*P* < 0.001
	I-KA group: T4 versus T1	*P* < 0.001	*P* < 0.001	*P* < 0.001	*P* < 0.001
	I-KA group: T3 versus T2	*P* < 0.001	*P* < 0.001	*P* < 0.001	*P* < 0.001
	I-KA group: T4 versus T2	*P* < 0.001	*P* < 0.001	*P* < 0.001	*P* < 0.001
	I-KA group: T4 versus T3	*P* = 0.242	*P* < 0.001	*P* < 0.001	*P* < 0.001

**Table 3:** Two-factor repeated measures ANOVA for the postoperative scores of patients diagnosed with type IV varus knee

Group	Time	VAS	KOOS	AROM	FJS-12 Knee
IV-MA (*n* = 32)	T1: preoperative	5.78 ± 1.31	48.13 ± 2.98	104.44 ± 3.29	
	T2: 6 weeks postoperative	4.52 ± 1.12	62.16 ± 3.89	110.31 ± 3.40	57.56 ± 2.99
	T3: 6 months postoperative	1.92 ± 1.06	70.59 ± 2.94	124.22 ± 3.13	69.91 ± 2.05
	T4: 1 year postoperative	1.32 ± 0.64	78.16 ± 3.03	132.75 ± 3.36	75.34 ± 1.56
IV-KA (*n* = 31)	T1: preoperative	5.84 ± 1.03	47.45 ± 3.12	103.45 ± 3.06	
	T2: 6 weeks postoperative	2.32 ± 1.23	70.55 ± 2.53	120.32 ± 2.70	58.29 ± 3.47
	T3: 6 months postoperative	1.87 ± 0.95	81.19 ± 2.51	125.10 ± 2.43	69.61 ± 2.59
	T4: 1 year postoperative	1.38 ± 0.72	90.00 ± 2.07	132.74 ± 3.02	87.13 ± 2.31
Overall comparison	HF correction factor				
	*F*, *P*-value between groups	31.649, *P* < 0.001	536.818, *P* < 0.001	48.323, *P* < 0.001	108.833, *P* < 0.001
	*F*, *P*-value within group	650.086, *P* < 0.001	1660.802, *P* < 0.001	977.172, *P* < 0.001	1363.193, *P* < 0.001
	Interaction *F*, *P*-value	33.312, *P* < 0.001	54.572, *P* < 0.001	41.047, *P* < 0.001	112.596, *P* < 0.001
Refined comparison between groups (*LSD-t* test, *P*-value)	T1	*P* = 0.304	*P* = 0.384	*P* = 0.224	
	T2	*P* < 0.001	*P* < 0.001	*P* < 0.001	*P* = 0.375
	T3	*P* = 0.477	*P* < 0.001	*P* = 0.219	*P* = 0.620
	T4	*P* = 0.136	*P* < 0.001	*P* = 0.992	*P* < 0.001
Refined comparison within group (*LSD-t* test, *P*-value)	IV-MA: T2 versus T1	*P* < 0.001	*P* < 0.001	*P* < 0.001	*P* < 0.001
	IV-MA: T3 versus T1	*P* < 0.001	*P* < 0.001	*P* < 0.001	*P* < 0.001
	IV-MA: T4 versus T1	*P* < 0.001	*P* < 0.001	*P* < 0.001	*P* < 0.001
	IV-MA: T3 versus T2	*P* < 0.001	*P* < 0.001	*P* < 0.001	*P* < 0.001
	IV-MA: T4 versus T2	*P* < 0.001	*P* < 0.001	*P* < 0.001	*P* < 0.001
	IV-MA: T4 versus T3	*P* = 0.267	*P* < 0.001	*P* < 0.001	*P* < 0.001
	IV-KA: T2 versus T1	*P* < 0.001	*P* < 0.001	*P* < 0.001	*P* < 0.001
	IV-KA: T3 versus T1	*P* < 0.001	*P* < 0.001	*P* < 0.001	*P* < 0.001
	IV-KA: T4 versus T1	*P* < 0.001	*P* < 0.001	*P* < 0.001	*P* < 0.001
	IV-KA: T3 versus T2	*P* < 0.001	*P* < 0.001	*P* < 0.001	*P* < 0.001
	IV-KA: T4 versus T2	*P* < 0.001	*P* < 0.001	*P* < 0.001	*P* < 0.001
	IV-KA: T4 versus T3	*P* = 0.001	*P* < 0.001	*P* < 0.001	*P* < 0.001

*VAS* Visual analog scale, *KOOS JR* Knee Injury and Osteoarthritis Outcome Score Joint Replacement, *AROM* Active range of motion, and *FJS-12 Knee* Forgotten Joint Score-12 Knee

## Discussion

Patients undergoing TKA using MA have reported postoperative pain and dissatisfaction with knee joint function [5–8]. In recent years, increasing research has focused on alternative alignment techniques for TKA, such as anatomical alignment, adjusted MA, KA, etc. Many studies have differentiated MA from KA; however, no research has assessed the different alignment methods used to treat the varying types of varus knees. Also patient-specifc instruments (PSI), computer-assisted surgery (CAS), or robotics are time-consuming and very expensive, which is difficult for our most patients’ families to afford, which makes it difficult to popularize advanced but expensive technologies [38–43]. Therefore, this study aimed to evaluate the subjective experiences and functional status of patients following TKA using the mechanical approach or KA technique with generic instruments for two distinct classes of varus knee. The basic clinical information, including sex, surgical side, age, and BMI, as well as the ASA grading were not markedly distinct between the two groups. This finding eliminates other factors besides surgery that could interfere with the study evaluation and results [44]. Our research further revealed no notable differences between the perioperative parameters, such as operation duration, intraoperative blood loss, and hospital stay length, suggesting that KA TKA does not increase surgical complexity or risk compared to MA TKA. Moreover, patients with mCPAK type I and IV varus knees did not experience significant differences in postoperative subjective sensations or knee joint function after undergoing surgery utilizing the same alignment technique. Thus, we did not distinguish between the two categories of varus knee in this research evaluation.

The focus of this study was to assess the postoperative subjective experiences and knee joint function of patients using three evaluation measures: the VAS, KOOS JR, and AROM. The VAS pain score reflects the patients’ subjective experiences, while the KOOS JR assesses the patients’ subjective feelings and postoperative knee joint function. Additionally, the AROM score mainly represents the patients’ knee joint function. In the initial 6 weeks after the surgery, the patients who underwent KA TKA had significantly lower VAS pain scores than those who received MA TKA, consistent with the results of the previous studies [19]. This difference could be because KA prevents overcorrection and ligament relaxation, which is required in MA. However, no notable differences were observed in the VAS pain scores between the two groups at the 6-month and 1-year follow-ups, possibly because pain tolerance gradually improves over time [45]. Furthermore, the patients undergoing KA TKA were revealed to have significantly higher scores on the KOOS JR than those with MA TKA at the 6-week, 6-month, and 1-year follow-up periods. This finding is in congruence with the previous research by Blakeney, which demonstrated that patients who underwent KA had knee joint kinematics closer to those of healthy individuals than those treated with the MA technique [12]. Moreover, many studies support this observation based on the notion that the KA protocol facilitates knee motion generation that is close to normal [18, 25, 46–48]. The KA technique provides a satisfactory compromise solution that allows the reconstruction of the anatomy of most patients while avoiding the overcorrection and ligament relaxation necessary for MA. Lastly, the AROM values indicated that the KA group elicited superior results compared to the MA group during the 6-week follow-up, in line with the previous study findings [12, 48]. However, no significant differences were detected in the AROM scores between the two alignment methods 6 months and 1 year after the surgery, potentially due to the patients’ improved rehabilitation response and adaptability [12, 48, 49].

Furthermore, we assessed the influence of the MA and KA methods on patients' postoperative subjective experiences via the FJS-12 Knee. At 1-year post-surgery, patients treated using the KA protocol had a significantly increased FJS-12 Knee, corresponding to the research findings by Howell [50, 51]. This observation may be attributed to the KA technique achieving close-to-normal knee kinematics and that most patients had a normally reconstructed anatomical structure [48]. Nonetheless, no notable differences were found in the FJS-12 Knee between the two alignment techniques at 6 months after the surgery, possibly because the patients had insufficient time to physiologically acclimate to the prosthetic joint following TKA. Correspondingly, an investigation by Carlson showed that the FJS-12 Knee of patients who underwent TKA was significantly lower at postoperative 6 months than 1 year after the surgery [23, 52].

In this study, we compared patients with mCPAK type I and IV varus knees who underwent unilateral TKA using the MA method versus those treated using the KA method. The grading system for varus knee (i.e., mCPAK classification) utilized in this study was proposed by Hsu et al. [33]. The classification method of mCPAK relies on the CPAK grading system developed by MacDessi et al. [32]. The mCPAK rectifies the uneven distribution when implementing the CPAK classification in Asian populations [32, 53, 54].

In preoperative planning, the alignment goal for each mCPAK classification type is more precisely set by incorporating the idea of aHKA angle, adjusted joint line orientation, and the concept of the restricted safe zone. Although the KA approach for type I and IV varus knees was proposed by Hsu et al., it was not clinically validated. Therefore, our research validates the employment of the KA method for individuals with type I and IV varus knee deformities, as defined by the mCPAK categorization. Moreover, our results indicated no significant differences in the postoperative subjective experiences or knee functionality of the patients with mCPAK type I or IV varus knees when undergoing surgery using the same alignment method (MA or KA). However, the limited number of patients enrolled in this study does not allow us to definitively conclude that a similar prognosis is obtained in patients with the two varus knee types who undergo the same alignment procedure. Thus, we plan to increase the sample size in further research studies.

Our study results inferred that compared to the MA method, the KA method greatly enhanced pain relief, motion range, and functionality in patients who underwent TKA, at least within the 1st year after the surgery. Our study suggests that patients with varus knee deformity may achieve greater benefits from the KA protocol, as evidenced by the improved patient-reported outcome measures (PROMs). However, our study has certain limitations that should be considered. First, we only used a posterior-stabilized design for knee prostheses. Although some researches have argued that a posterior-stabilized implant is no worse than a medially stabilized implant when applied to KA techniques, many experts do apply a medially stabilized implant with unrestricted or restricted KA; thus, the generalizability of our findings to other implant designs remains uncertain [55, 56]. Second, our investigation was focused on patients with varus knee deformity. Thus, our findings may not apply to valgus knee cases. Third, the number of patients included in this study was small. We hope to address this issue by increasing the sample size in the future investigations. Finally, this study was limited to a 1-year postoperative follow-up of the patients, focusing on their clinical outcome measures. Therefore, extended follow-up is essential to acquire long-term clinical evaluation.

## Conclusion

Our study revealed that the KA method led to significant enhancements in pain relief, range of motion, and function compared to the MA method within the 1st postoperative year in patients undergoing TKA surgery. Furthermore, the KA procedure did not differ from the MA method in terms of surgical difficulty or risk. Therefore, the KA technique may be considered a strategic approach when selecting a suitable TKA implant with generic instruments for individuals with type I or IV varus knees.

## Typical cases

*Case 1* Patient, female, 63 years old, type I knee varus, treated with MA TKA (Fig. 1).

*Case 2* Patient, female, 68 years old, type I knee varus, treated with KA TKA (Fig. 2).

*Case 3* Patient, male, 61 years old, type IV knee varus, treated with MA TKA (Fig. 3).

*Case 4* Patient, female, 78 years old, type IV knee varus, treated with KA TKA (Fig. 4).
